# DeepCCI: a deep learning framework for identifying cell–cell interactions from single-cell RNA sequencing data

**DOI:** 10.1093/bioinformatics/btad596

**Published:** 2023-09-23

**Authors:** Wenyi Yang, Pingping Wang, Meng Luo, Yideng Cai, Chang Xu, Guangfu Xue, Xiyun Jin, Rui Cheng, Jinhao Que, Fenglan Pang, Yuexin Yang, Huan Nie, Qinghua Jiang, Zhigang Liu, Zhaochun Xu

**Affiliations:** School of Life Science and Technology, Harbin Institute of Technology, Harbin 150006, China; School of Life Science and Technology, Harbin Institute of Technology, Harbin 150006, China; School of Life Science and Technology, Harbin Institute of Technology, Harbin 150006, China; School of Life Science and Technology, Harbin Institute of Technology, Harbin 150006, China; School of Life Science and Technology, Harbin Institute of Technology, Harbin 150006, China; School of Life Science and Technology, Harbin Institute of Technology, Harbin 150006, China; School of Life Science and Technology, Harbin Institute of Technology, Harbin 150006, China; School of Life Science and Technology, Harbin Institute of Technology, Harbin 150006, China; School of Life Science and Technology, Harbin Institute of Technology, Harbin 150006, China; School of Life Science and Technology, Harbin Institute of Technology, Harbin 150006, China; School of Life Science and Technology, Harbin Institute of Technology, Harbin 150006, China; School of Life Science and Technology, Harbin Institute of Technology, Harbin 150006, China; School of Life Science and Technology, Harbin Institute of Technology, Harbin 150006, China; Affiliated Foshan Maternity & Child Healthcare Hospital, Southern Medical University, Guangzhou 510515, China; School of Life Science and Technology, Harbin Institute of Technology, Harbin 150006, China

## Abstract

**Motivation:**

Cell–cell interactions (CCIs) play critical roles in many biological processes such as cellular differentiation, tissue homeostasis, and immune response. With the rapid development of high throughput single-cell RNA sequencing (scRNA-seq) technologies, it is of high importance to identify CCIs from the ever-increasing scRNA-seq data. However, limited by the algorithmic constraints, current computational methods based on statistical strategies ignore some key latent information contained in scRNA-seq data with high sparsity and heterogeneity.

**Results:**

Here, we developed a deep learning framework named DeepCCI to identify meaningful CCIs from scRNA-seq data. Applications of DeepCCI to a wide range of publicly available datasets from diverse technologies and platforms demonstrate its ability to predict significant CCIs accurately and effectively. Powered by the flexible and easy-to-use software, DeepCCI can provide the one-stop solution to discover meaningful intercellular interactions and build CCI networks from scRNA-seq data.

**Availability and implementation:**

The source code of DeepCCI is available online at https://github.com/JiangBioLab/DeepCCI.

## 1 Introduction

Multicellular life relies on the conformity of cellular activities, which depend on cell–cell interactions (CCIs) across diverse cell types (Zhou *et al.* 2018, Giladi *et al.* 2020). Single-cell RNA-sequencing (scRNA-seq) technologies have enabled remarkable progress in understanding cellular mechanisms at an unprecedented resolution level (Yuan *et al.* 2017, Gao *et al.* 2018). Although scRNA-seq data inherently contains gene expression information that could be used to identify intercellular communications, it remains a great challenge to explore potential CCIs that often drive heterogeneity and cell state transitions (Jin *et al.* 2021). The signaling events behind cells are usually mediated by interactions of various types of proteins, encompassing ligand–receptor (L–R), receptor–receptor, and extracellular matrix–receptor interactions. Especially, multi-subunit L–R complexes are critical for CCIs (Jl 1992, Efremova *et al.* 2020). Some proteins, such as TGF-beta (transforming growth factor-beta) receptors10 and cytokine receptors (Sato and Miyajima 1994), require multi-subunit assembly for function (Armingol *et al.* 2021). Specifically, in the TGF-beta signaling pathway, the interaction between soluble ligand TGFB1 and the heteromeric complexes of type I and type II receptors (TGFBR1 and TGFBR2) plays an important role in the development of diabetic nephropathy (McKnight *et al.* 2007).

To identify CCIs from scRNA-seq data, several computational strategies have been developed based on the L–R gene pairs, such as SingleCellSignalR (Cabello-Aguilar *et al.* 2020) iTALK (Wang *et al.* 2019), CellPhoneDB, and CellChat. Each strategy consists of a resource of intercellular interactions prior knowledge and a method to identify underlying CCI events. However, the identified results of these strategies are usually limited by the comprehensiveness of the prior L–R gene pair database. Different L–R pair databases used in each method could contribute to the variety of identified interactions. Also, identifying previously uncharacterized cell types in heterogeneous scRNA-seq data is the precondition for identifying CCIs (Plasschaert *et al.* 2018, Suo *et al.* 2018). Nevertheless, these methods cannot classify cells into cell clusters independently before interaction identification. Moreover, due to the technical difficulties of capturing single-cell proteomic information at present, defining the ground truth of CCI networks is challenging. Recently, deep learning-based methods have demonstrated their prowess in a broad range of single-cell studies. However, there is still no deep learning framework for CCI prediction from scRNA-seq data. Combining scRNA-seq data with deep learning technologies will be greatly expanded to provide unique insights into CCI prediction.

Here, we develop DeepCCI, a graph convolutional network (GCN) (Bo *et al.* 2020)-based deep learning framework (https://github.com/JiangBioLab/DeepCCI) for CCI identification from scRNA-seq data. To explore the interactions between cells from scRNA-seq data in one-stop, DeepCCI provides two deep learning models: (i) a GCN-based unsupervised model for cell clustering, and (ii) a GCN-based supervised model for CCI identification. DeepCCI has great potential to cluster cells and capturing biological meaningful interactions between cell clusters by utilizing the underlying complex gene expression patterns of heterogeneous cells from scRNA-Seq data. DeepCCI first learns an embedding function that jointly projects cells into a shared embedding space using Autoencoder (AE) (Eraslan *et al.* 2019) and GCN. By using the embedding information, DeepCCI clusters cells into several groups. And then, we manually curated a comprehensive signaling-molecule interaction database termed LRIDB for L–R interactions with multi-subunits. According to LRIDB, DeepCCI predicts intercellular crosstalk between any pair of clusters within a given scRNA-seq data. Also, DeepCCI provides several visualization outputs to show how strongly or specifically each cell cluster interacts with every other cell cluster. We demonstrate the overall capabilities of DeepCCI by applying it to several publicly available scRNA-seq datasets. The results indicated that DeepCCI has excellent potential in capturing biological relationships between cells in terms of cell-type clustering and CCI prediction from scRNA-seq data.

## 2 Materials and methods

### 2.1 Database construction for ligand–receptor pairs

The availability of a comprehensive set of signaling L–R pairs is crucial for accurate prediction of biologically meaningful intercellular communications. In recent years, numerous L–R pair databases [such as Baccin2020, Cain2020 (Cain *et al.* 2020), CellChatDB, CellCallDB (Zhang *et al.* 2021), CellPhoneDB, LRdb (Cabello-Aguilar *et al.* 2020), connectomeDB2020 (Hou *et al.* 2020), CellTalkDB (Shao *et al.* 2021), and iTALK] have emerged with the goal of predicting CCIs. These databases provide literature-supported L–R pairs for both human and mouse.

To construct a database that represents the current state of knowledge on L–R interactions, we manually reviewed publicly available L–R interaction databases and developed LRIDB. LRIDB encompasses L–R interactions in both mouse and human. In order to compile LRIDB, we carefully curate a collection of L–R pairs by manually selecting from these databases. Our selection process is focused on acquiring a comprehensive and exhaustive set of L–R pairs.

Firstly, we prioritize interactions associated with literature sources to establish a strong foundation of reliable and well-documented L–R interactions in LRIDB. Subsequently, we have removed redundant L–R pairs and retain interactions that provide the most comprehensive information. In addition, we take into account the biological relevance of predicting interactions by considering the existence of multi-subunit complexes. These complexes serve as vital functional units in cellular processes and often involve intricate interactions between ligands and receptors. Consequently, we integrate these complex structures into our analysis and exclude any duplicate ligands or receptors associated with them. As a result, LRIDB preserves 5127 validated L–R interactions for human and 4623 for mouse (Supplementary Data S1 and S2).

### 2.2 Dataset preprocessing

DeepCCI takes the scRNA-Seq gene expression profile as the input. Data filtering and quality control are the first steps of data preprocessing, only genes expressed as nonzero in more than 1% of cells, and cells expressed as nonzero in more than 1% of genes are kept. For the prediction of cell clusters, we used Seurat to normalize each cell to 10 000 read counts and log-transformed the data. Then, genes are ranked by standard deviation, i.e. the top 2000 genes in variances are used for the study. For the study of the interactions between cell clusters, we adopted another different data processing method. First, we identified differentially expressed signaling genes across all cell clusters within a given scRNA-seq dataset, using the Wilcoxon rank sum test (Wilcoxon 1946) with a significance level of 0.05. Next, we project gene expression data onto a high-confidence experimentally validated protein–protein interaction (PPI) network from STRINGdb (Ronen and Akalin 2018). Overexpressed L–R pairs were selected if a protein of each interaction was in the list of ortholog ligands and the other was in the list of ortholog receptors. The obtained gene expression data are used for the definition and prediction of interaction between cell clusters. To reduce the effects of the extreme values, we calculated the ensemble average expression of ligands and receptors in a given cell cluster using a truncated mean method and 10% was selected as the truncation ratio.

### 2.3 Autoencoder (AE) and graph convolutional network (GCN)

The basic AE is used to learn the representations of the single-cell expression data in order to accommodate for different kinds of data characteristics. We assume that there are *L* layers in the AE and *l* represents the layer number. Specifically, the representation learned by the *l*th layer in encoder part, $H^{\left(l\right)}$, can be obtained as follows:

(1) $$H^{\left(l\right)}=\phi (W_{e}^{\left(l\right)}H^{\left(l-1\right)}+b_{e}^{\left(l\right)}),$$

where ϕ is the activation function of the fully connected layers, $W_{e}^{\left(l\right)}$ and $b_{e}^{\left(l\right)}$ are the weight matrix and bias of the *l*th layer in the encoder, respectively. Besides, we denote $H^{\left(0\right)}$ as the preprogressed single-cell expression data X.

The encoder part is followed by the decoder part, which is to reconstruct the input data through several fully connected layers by the equation:

(2) $$H^{\left(l\right)}=\phi (W_{d}^{\left(l\right)}H^{\left(l-1\right)}+b_{d}^{\left(l\right)}),$$

where $W_{d}^{\left(l\right)}$ and $b_{d}^{\left(l\right)}$ are the weight matrix and bias of the *l*th layer in the decoder.

The output of the decoder part is the reconstruction of the feature data X, which results in the following objective loss function:

(3) $$L_{\mathrm{res}}=\frac{1}{2N}\sum_{i=1}^{N}||X-\hat{X}|{|}_{F}^{2},$$

where $\hat{X}$ is the reconstructed expression data by the AE.

The GCN is used to explore the underlying topology information of the cell graph and L–R pair graph. The GCN module is used to accommodate two different kinds of information, i.e. data itself and relationship between data. The essential idea is to update the node representations by propagating information between nodes. With the weight matrix W, the representation learned by the *l*th layer of GCN, $Z^{\left(l\right)}$, can be obtained by the following convolutional operation:

(4) $$Z^{\left(l\right)}=\varphi ({\tilde{D}}^{-\frac{1}{2}}\tilde{A}{\tilde{D}}^{-\frac{1}{2}}Z^{\left(l-1\right)}W^{\left(l-1\right)}),$$

where $\tilde{A}=A+I$ and ${\tilde{D}}_{ii}=\sum_{j}{\tilde{A}}_{ij}$. I is the identity diagonal matrix of the adjacent matrix A for the self-loop in each node. The representation $Z^{\left(l-1\right)}$ will propagate through the normalized adjacency matrix ${\tilde{D}}^{-\frac{1}{2}}\tilde{A}{\tilde{D}}^{-\frac{1}{2}}$ to obtain the new representation $Z^{\left(l\right)}$.

### 2.4 Model construction for single-cell clusters

To infer the cluster of single cells, we first built the cell graph from a KNN graph, where nodes are individual single cells, and the edges are relationships between cells. *K* (default = 10) is the predefined parameter used to control the scale of the captured interaction between cells. Each node finds its neighbors within the *K* shortest distances and creates edges between them and itself.

Considering that the representation learned by AE $H^{\left(l\right)}$ is able to reconstruct the feature itself and contains different valuable information, we combine the two representations $Z^{\left(l\right)}$ and $H^{\left(l\right)}$ together to get a more complete and powerful representation as follows:

(5) $${\tilde{Z}}^{\left(l\right)}=(1-\varepsilon )Z^{\left(l\right)}+\varepsilon H^{\left(l\right)},$$

where ε is a coefficient to balance the weights of GCN and AE, and we uniformly set it to 0.5 here. In this way, we connect the AE and GCN layer by layer to get the more informative embedding for the next clustering progress.

Then we use ${\tilde{Z}}^{\left(l\right)}$ as the input of the *l*th layer in GCN to generate the representation ${\tilde{Z}}^{\left(l+1\right)}$:

(6) $$Z^{\left(l+1\right)}=\phi ({\tilde{D}}^{-\frac{1}{2}}\tilde{A}{\tilde{D}}^{-\frac{1}{2}}{\tilde{Z}}^{\left(l\right)}W^{\left(l\right)}),$$

the AE-specific representation $H^{\left(l\right)}$ will be propagated through the normalized adjacency matrix ${\tilde{D}}^{-\frac{1}{2}}\tilde{A}{\tilde{D}}^{-\frac{1}{2}}$. To preserve information as much as possible, we transfer the representations learned from each GCN layer into a corresponding encoder layer and decoder layer for information propagation. Now, we have connected the AE with GCN in the neural network architecture. Here, we unify the AE and GCN modules in a uniform framework and effectively trains the two modules end-to-end for clustering. Learning an effective data representation is of great importance to deep clustering. Pretraining is a required step of the DeepCCI model, and it gives a substantial boost in the performance compared to starting from the random weights.

In particular, for the *i*th cell and *j*th cluster, we use the Student’s *t*-distribution as a kernel to measure the similarity between the data representation hi and the cluster center vector *µ_j_* as follows:

(7) $$q_{ij}=\frac{(1+||h_{i}-\mu_{j}|{|}^{2}/v{)}^{-\frac{v+1}{2}}}{\sum_{j\prime }(1+||h_{i}-\mu_{j^{\prime }}|{|}^{2}/v{)}^{-\frac{v+1}{2}}},$$

where *h_i_* is the *i*th row of H^(L)^, *μ_j_* is initialized by *K*-means on representations learned by pretrained AE and *v* are the degrees of freedom of the Student’s *t*-distribution. *q_ij_* can be considered as the probability of assigning cell *i* to cluster *j*. We treat *Q* = [*q_ij_*] as the distribution of the assignments of all samples and let *α* = 1 for all experiments.

After obtaining the clustering result distribution *Q*, we aim to optimize the data representation by learning from the high confidence assignments. Specifically, we want to make data representation closer to cluster centers, thus improving the cluster cohesion. Hence, we calculate a target distribution *P* as follows:

(8) $$p_{ij}=\frac{q_{ij}^{2}/f_{j}}{\sum_{j\prime }q_{ij^{\prime }}^{2}/f_{j^{\prime }}},$$

where $f_{j}=\sum_{i}q_{ij^{}}$ are soft cluster frequencies. In the target distribution *P*, each assignment in *Q* is squared and normalized so that the assignments will have higher confidence, leading to the following objective function:

(9) $$L_{\mathrm{clu}}=KL(P||Q)=\sum_{i}\sum_{j}p_{ij}\log\frac{p_{ij}}{q_{ij^{}}},$$

By minimizing the KL divergence loss between *Q* and *P* distributions, the target distribution *P* can help the encoder learn a better representation to make the data representation surrounding the cluster centers closer. The target distribution *P* is calculated by the distribution *Q*, and the *P* distribution supervises the updating of the distribution *Q* in turn.

The GCN module will also provide a clustering assignment distribution *Z*. Therefore, we can use distribution *P* to supervise distribution *Z* as follows:

(10) $$L_{\mathrm{gcn}}=KL(P||Z)=\sum_{i}\sum_{j}p_{ij}\log\frac{p_{ij}}{z_{ij^{}}},$$

The overall loss function of the cluster model is:

(11) $$L=\alpha L_{\mathrm{clu}}+\beta L_{\mathrm{gcn}}+L_{\mathrm{res}},$$

α , β and θ are used to balance the contribution of AE and GCN. In this study, we set α = 0.0001, β = 0.001 for all datasets. Finally, we choose the soft assignments in distribution *Q* as the final clustering results. The label assigned to cell *i* is:

(12) $$r_{i}=\underset{j}{\mathrm{argmax}}(q_{\text{ij}}),$$

### 2.5 Identification of statistically significant interactions

We calculate the interaction probability value of L–R mediated cluster–cluster interactions by using the expression product of two cell clusters. Based on the projected data progressed, the interaction probability *P_i,j_* from cell clusters *i* to *j* for a particular L–R pair k was modeled by:

(13) $$P_{i,j}^{k}=L_{i}R_{j}=\sqrt[{m1}]{L_{i,1}\cdot \cdot \cdot L_{i,m1}}\times \sqrt[{m2}]{R_{j,1}\cdot \cdot \cdot R_{j,m2}},$$

where *L_i_* and *R_j_* represent the average expression level of ligand L and receptor R in cell group *i* and cell group *j*, respectively. The expression level of ligand L with m1 subunits (i.e. *L_i_*_,1_, ⋯, *L_i,m_*_1_) was approximated by their geometric mean, implying that the zero expression of any subunit leads to an inactive ligand. Similarly, we computed the expression level of receptor R with *m*2 subunits.

Then, the significant interactions between two cell clusters are identified using the publicly available statistical methods (CellChat, CellPhoneDB, SingleCellSignalR). Specifically, we applied these methods to the single-cell datasets by using the same L–R interactions database, LRIDB, to identify the statistically significant interactions. By using the threshold (LRscore ≥ 0.5 or *P*-value ≤ 0.01) for each method, we selected the statistically significant interactions as the true labels of the model. Specifically, to explore more meaningful interactions, the majority vote was used to select the significant interactions.

### 2.6 Model construction for single-cell CCIs

Residual Network (ResNet) and GCN were used to build the interaction model of DeepCCI. The input of ResNet is the expression values of ligands in the source cell cluster and those of receptors in the target cell cluster. To fully utilize the information of each cell, we constructed the feature using the expression of the ligand of all cells in source cell cluster and the expression of the receptor of all cells in target cell cluster. To embed subunit information in deep learning, the geometric mean was applied to process the multi-subunit ligands or receptors present in each cell. To normalize feature data and improve model prediction speed, we use Singular Value Decomposition (SVD) (de Leeuw 2006) method to reduce the feature data dimension before inputting the deep learning model. Simultaneously, the GCN model of this part receives the L–R pair graph that was constructed by the progressed scRNA-seq data and LRIDB. The nodes of L–R pair graph are the highly expressed L–R pairs in the scRNA-seq data, with the edges representing the relationships between these pairs. The highly expressed L–R pairs are defined based on the interaction probability. Specifically, we calculated the sum of the interaction probability of each L–R of all CCIs and selected the top 200 L–R pairs to construct the relationship between them. The correlation matrix *P* of all L–R pairs is built based on the Pearson correlation coefficient between each pair. Specifically, we use the threshold τ (default: 0.95) to filter edges, and the adjacent matrix ALR of L–R pairs can be written as:

(14) $$A_{ij}^{LR}=\left\{\begin{array}{c} 0,if\begin{array}{cc} P_{ij}\geq \tau & \end{array}\\ 1,if\begin{array}{cc} P_{ij}<\tau & \end{array} \end{array}\right.,$$

where $A_{ij}^{LR}$ is the binary correlation matrix. In this way, we can capture the correlations between L–R pairs and explore these correlations to improve the classification performance for the interactions between two cell clusters. And then, the output of the ResNet and the GCN are applied by dot product for the following fully connected layer. The FC layers are used to predict L–R interactions with the cluster state. We assume that the ground-truth label of a significant interaction is y, where yi denotes whether label i represent the significant interaction. The whole network is trained using the Focal loss (Lin *et al.* 2017) as follows:

(15) $$L_{fl}=\left\{\begin{array}{c} -(1-y\prime {)}^{\gamma }\log\;y\prime ,y=1\\ -y\prime^{\gamma }\log(1-y\prime ),y=0 \end{array}\right.,$$

where y′ is the output of the FC layers activated by the sigmoid function.

### 2.7 The evaluation criteria for the DeepCCI

Adjusted Rand Index (ARI) is used to compute similarities by considering all pairs of the cells that are assigned in clusters in the current and previous clustering adjusted by random permutation:

(16) $$\mathrm{ARI}=\frac{RI-E[RI]}{\max(RI)-E[RI]},$$

where the unadjusted rand index (RI) is defined as:

(17) $$RI=\frac{a+b}{C_{n}^{2}},$$

where *a* is the number of pairs correctly labeled in the same sets, and *b* is the number of pairs correctly labeled as not in the same dataset. $C_{n}^{2}$ is the total number of possible pairs. E[RI] is the expected RI of random labeling.

The performance of DeepCCI on the prediction of CCIs was evaluated by using recall, precision, accuracy (ACC) and F1-score, which are well-known and have been widely used in the field of bioinformatics, defined as:

(18) $$\text{recall}=\frac{\text{TP}}{\text{TP}+\text{FN}},$$

 

(19) $$\;\text{precision}=\frac{\text{TP}}{\text{TP}+\text{FP}},$$

 

(20) $$\text{ACC}=\frac{\text{TP}+\text{TN}}{\text{TP}+\text{FP}+\text{TN}+\text{FN}},$$

 

(21) $$F_{1}=2\cdot \frac{\mathrm{precision}\cdot \mathrm{recall}}{\mathrm{precision}+\mathrm{recall}},$$

where TP represents true positive; TN, true negative; FP, false positive; FN, false negative.

The area under the curve (AUC) is an important metric of performance evaluation of the proposed model, defined as the area under the Receiver Operating Characteristic Curve (ROC) that can be used for visualizing the performance of interaction prediction.

### 2.8 Method comparisons of DeepCCI cluster model

To evaluate the cell clustering performance of DeepCCI, we compared the cell cluster model of DeepCCI with several state-of-the-art methods [scTAG, Graph-sc, scGNN, scGAE, GraphSCC, scziDesk, scDCC, DCA, DEC, K-means, Spectral, Seurat(Louvain), Seurat(Leiden)] on 12 real-world scRNA-seq datasets (the detailed information is described in Supplementary Data S3) from several representative sequencing platforms. The widely used clustering metrics ARI was used to measure the clustering performance of cluster model of DeepCCI and the other 13 baseline methods. To avoid the error caused by chance, each clustering method was run 10 times to take the average.

### 2.9 Datasets used to evaluate the interaction model of DeepCCI

To construct an interaction identification model with strong generalization, a published integrated pancreatic islets scRNA-seq dataset (panc8) (Butler *et al.* 2018) across multiple platforms was used. The labeled panc8 data contains eight pancreas datasets across five technologies (CEL-Seq, CEL-Se2, Fluidigm C1, SmartSeq2, inDrops) integrated and normalized with the R toolkit Seurat. The panc8 dataset includes 14 892 cells with 13 benchmark cell labels [Acinar, activated stellate(aPSC), Alpha, Beta, Delta, Ductal, Endothelial, Epsilon, Gamma, Macrophage, Mast, quiescent stellate(aPSC), Schwann] (Fig. 3a). To capture the high-confident interactions between cell clusters, we use the benchmark cell labels of the panc8 dataset for further analysis. Based on the LRIDB, we calculate the interaction probability of L–R pairs for the cell cluster pairs and define the significant L–R pairs by using three statistical methods.

The human atopic dermatitis (AD) dataset and embryonic mouse skin dataset were utilized as independent test sets for the purpose of evaluation. The human AD dataset contains 17 349 cells which were clustered into 12 cell groups, including T cells (TC), APOE + fibroblast (FIB), CD40LG + TC, COL11A1+FIB, FBN1+FIB, inflammatory dendritic cells (Inflam.DC), Inflam.FIB, Inflam.TC, Langerhans cells (LC), Natural Killer T cells (NKT), cDC1, and cDC2. The cell clusters for the two datasets are visualized using the UMAP method. The embryonic mouse skin dataset contains 25 148 cells, which were clustered into 13 cell groups, including Basal, Basal-P, DC, endothelial (ENDO), FIB-A, FIB-B, FIB-P, Immune, melanocyte (MELA), myeloid (MYL), Muscle, Pericyte, and Spinious. To verify the generalizability of DeepCCI interaction model independently, we used the benchmarked cell labels of these two datasets to predict significant CCIs.

### 2.10 Method comparisons of DeepCCI interaction model

We compare the performance of DeepCCI with seven other methods, including SingleCellSignalR, iTALK, CellPhoneDB, CellChat, CellCall, CytoTalk, and NATMI. SingleCellSignalR uses a regularized expression product to compute L–R interaction scores and is the only tool reviewed that provides explicit cut-off values for this score to achieve appropriate false discovery rates based on empirical results. iTALK uses downstream analysis methods to curate the final list of significantly differentially expressed genes between cell clusters in single-cell RNA-seq data are identified, and these lists are analyzed for L–R pairs. CellCall and CytoTalk use known interactions between ligands, receptors and downstream targets to build a network of L–R relationships. Lists with multimeric proteins are used in CellPhoneDB and CellChat to assess whether all subunits are simultaneously expressed to identify likely functional L–R interactions. All the methods were applied to infer intercellular communications using their own default parameters.

To demonstrate the performance of DeepCCI from multiple aspects, we use three datasets from different techniques [a scRNA-seq dataset of human testicular (Wang *et al.* 2018), a seqFISH dataset of mouse organogenesis and 10× Visium spatial transcriptomics dataset of coronal section of the mouse brain (Villacampa *et al.* 2020)].

For the scRNA-seq dataset of human testicular, the curated literature related CCIs from Sertoli cells (STs) to spermatogonial stem cells (SSCs) are provided as the ground truth. The area under curve of receiver operating characteristic (AUC) and precision were used as evaluation criteria.

Next, we leveraged spatial information as a way to support the predictive potential of DeepCCI, under the assumption that spatially adjacent cell types are expected to have stronger CCI than other nonadjacent cell types. To more intuitively show the interaction differences between cell types at different distances, five cell types with obvious clustering were selected in this case, including Lateral plate mesoderm (LPM), Forebrain/Midbrain/Hindbrain (FMH), Spinal cord (SC), Splanchnic mesoderm (SM), and Cardiomyocytes (Card). We calculated the average distance between these cell types and selected the FMH as the source cell type and computed the interaction probabilities or scores between FMH and other cell types (Supplementary Fig. S6).

For the 10× Visium spatial transcriptomics dataset, five cell types were selected to study the interactions between cell clusters, including Cortex_1 (C1), Cortex_3 (C3), Cortex_4 (C4), Hypothalamus_1 (H1), and Hypothalamus_2 (H2). The average distance between these cell types were calculated (Supplementary Fig. S7). The C4 cell type was selected as the source cell type and the interaction probabilities or scores from C4 cell type to other cell types were computed.

### 2.11 *De novo* prediction of DeepCCI

To test the complete ability of DeepCCI for cell clustering and interaction prediction, we applied DeepCCI on a scRNA-seq dataset called PBMC3k (Zheng *et al.* 2017) generated using 10× Genomics technology. PBMC3k contains 2700 cells with 9 benchmarked labels. To display the predicted cell clusters more intuitively, the scCATCH (Shao *et al.* 2020) was used to labeled the predicted cell clusters.

## 3 Results

### 3.1 Overview of the DeepCCI workflow

**Figure 1.**
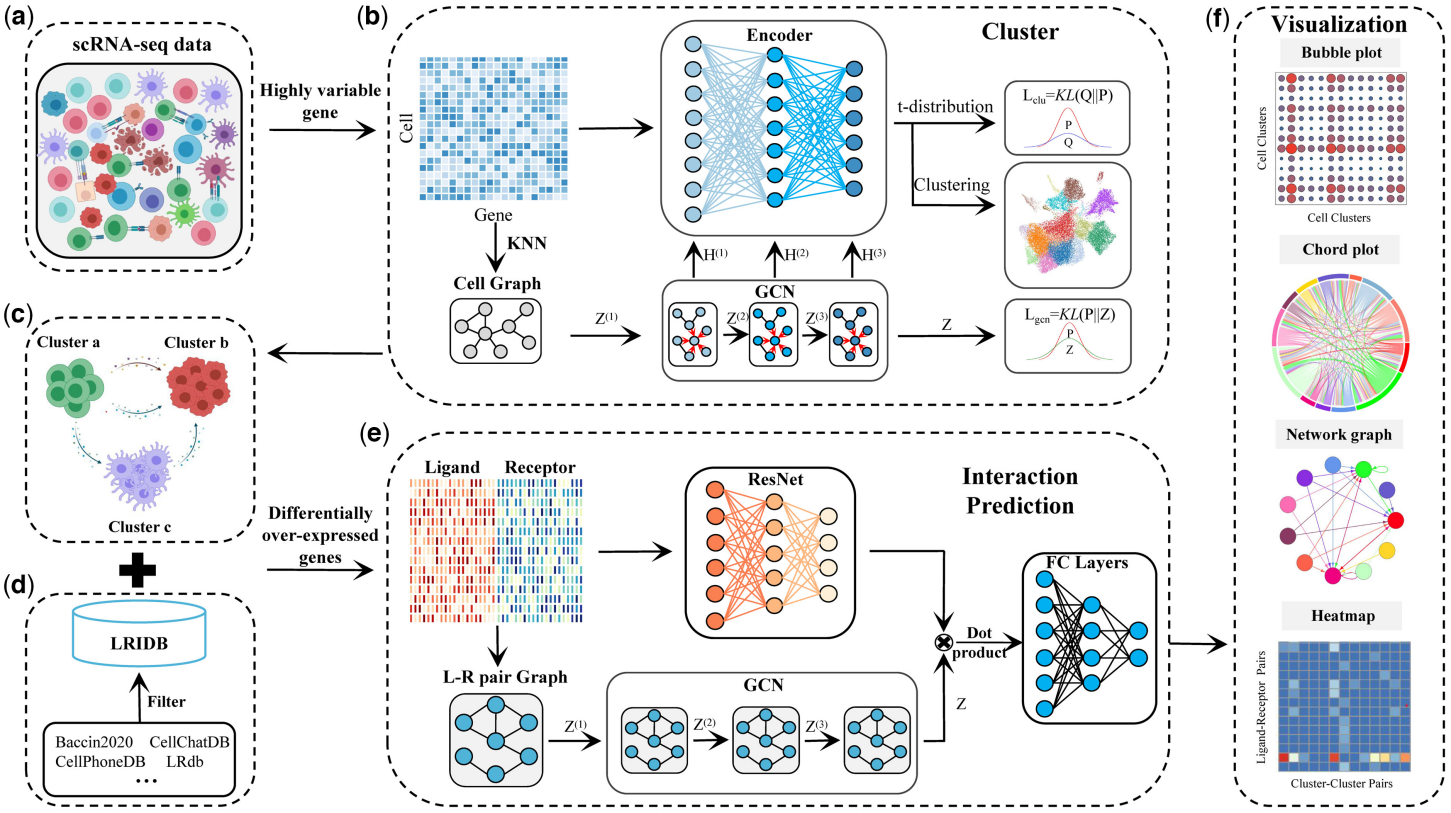
Workflow of the DeepCCI. (a) DeepCCI takes the scRNA-seq data as input. (b) DeepCCI clusters cells using the AE and the GCN jointly. (c) ScRNA-seq data with cell types. (d) LRIDB contains validated L–R interactions that were collected from several publicly literature-supported databases. (e) DeepCCI predicts the interactions between cell clusters using ResNet and GCN jointly. (f) DeepCCI offers several visualization outputs for different analytical tasks

Given scRNA-seq data, DeepCCI seeks influential representations of cells helpful in performing different tasks in scRNA-seq data analyses (Fig. 1). DeepCCI consists of two major functions: (i) clustering cells from scRNA-seq data, and (ii) establishing interaction networks between cell clusters with prior knowledge of signaling L–R pairs.

Cell clustering is a prerequisite for CCI identification, different number of cell clusters may naturally affect the identified interactions. To establish CCI networks, DeepCCI can operate in label-based and label-free modes. In its label-based mode, DeepCCI requires user-assigned cell labels as the input. In its label-free mode, DeepCCI provides an unsupervised clustering model by using AE and GCN jointly to cluster cells (Fig. 1b). The cluster model intakes the gene expression matrix after data preprocessing, including low-quality cells and genes removing, normalization, and variable gene ranking (Hu *et al.* 2020, Menden *et al.* 2020). To achieve better cluster performance, we pretrained an AE model using the top 2000 variable genes (Zhong *et al.* 2021). By minimizing a data reconstruction error, the pretrained AE learns a low-dimensional embedding to reconstruct the gene expression matrix. The pretraining step serves as a prior for the parameter space, which is helpful for the subsequent cluster progress. Then, the pretrained AE was combined with the GCN to build the cluster model. The input of GCN is the cell graph, which is generated from the top 2000 variable gene expression matrix using the KNN algorithm. The nodes of cell graph represent individual cells while the edges represent neighborhood relations among these cells (Li *et al.* 2021, Wang *et al.* 2021, Zheng *et al.* 2021). Next, the cluster model regenerates the whole graph structure and learns a low-dimensional embedding of each cell jointly using AE and GCN. Based on the learned space embedding, the k-means clustering method was used to cluster cells (Xu and Lange 2019, Baccin *et al.* 2020).

The more comprehensive set of the signaling L–R pairs is of vital importance to prediction of biologically meaningful intercellular communications. To investigate the significant interaction between cell clusters, we first identified differentially over-expressed ligands and receptors for each cell cluster based on LRIDB. Next, we associated each interaction with a probability value to quantify interactions between two cell clusters. Specifically, we calculated the interaction probability based on the average expression values of a ligand and the cognate receptor. Three publicly available statistical methods were used to identify the significant interactions (*P*-value < 0.01 or LRscore > 0.5). We set these significant interactions as positive samples and the remaining interactions with probability values as negative samples for model training (see Section 2). Subsequently, we built a deep learning model for accurately predicting significant CCIs jointly using the Residual Network (ResNet) and GCN (Fig. 1e). The GCN model of this part receives the L–R pair graph that was constructed by the progressed scRNA-seq data and LRIDB. The nodes of L–R pair graph are the L–R pairs, with the edges representing the relationships between these pairs. Simultaneously, the input of ResNet is the expression values of ligands in one cell cluster and those of receptors in another cell cluster. The dot product of outputs from ResNet and GCN was fed into the next fully connected layers and the significant interactions between cell clusters were predicted (see Section 2). In addition, several informative and intuitive visualization methods were used to display the significant connections between interacting cell clusters (Fig. 1f).

### 3.2 Performance evaluation of cluster model of DeepCCI

**Figure 2.**
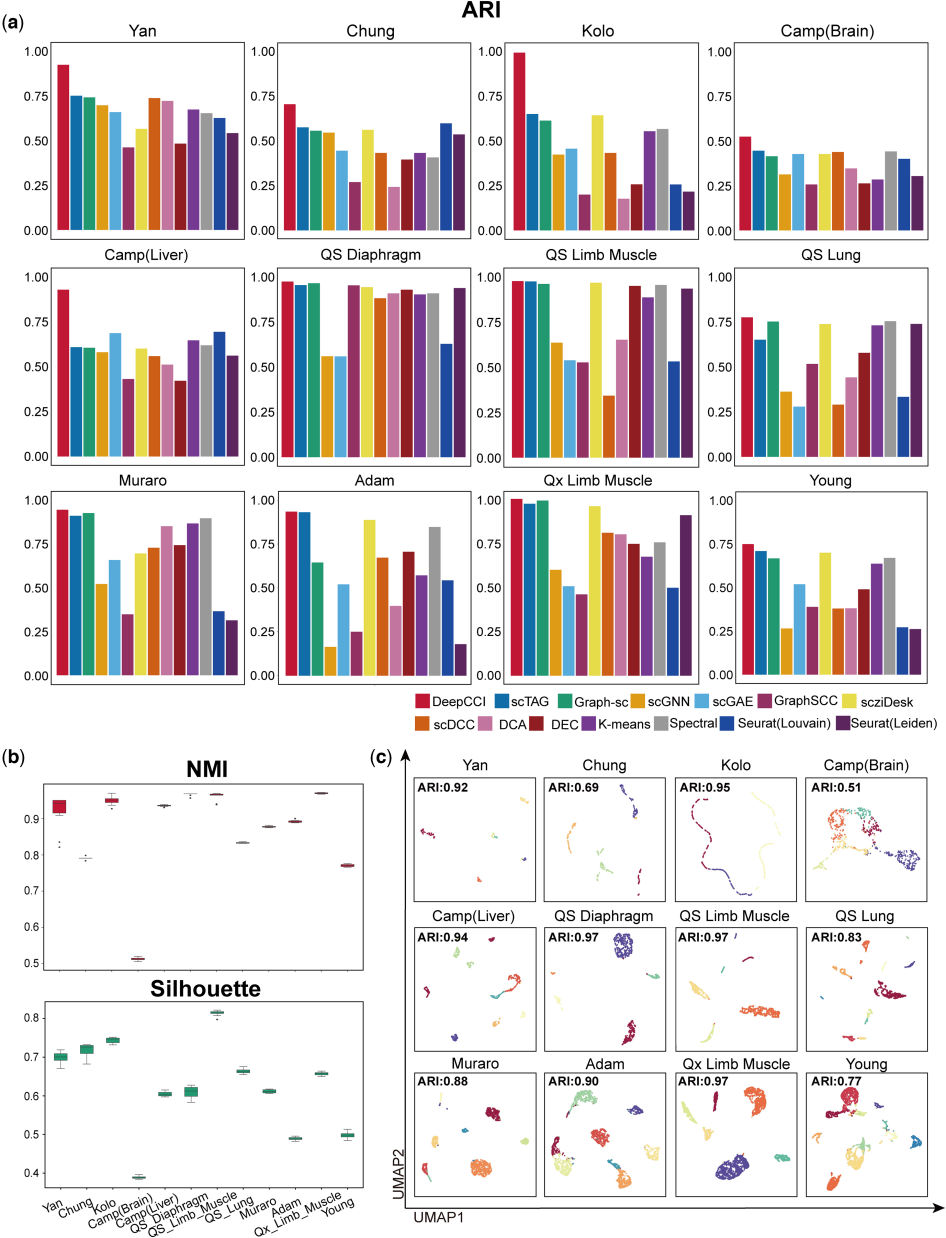
Performance evaluation of cell cluster model of DeepCCI. (a) Comparison of ARI among cell cluster model of DeepCCI and 13 state-of-the-art methods. (b) Performance evaluations of the cell clustering by repeating cell cluster model of DeepCCI 10 times. (c) UMAP visualizations for 12 scRNA-seq datasets using the cell embedding generated by cell cluster model of DeepCCI

We compared the cell clustering results of DeepCCI with several scRNA-Seq analytical frameworks. For the 12 scRNA-seq datasets, the cluster model of DeepCCI achieved the best ARI on all of them, and even in “Qx Limb Muscle,” the ARI reached 0.9848 (Fig. 2a, Supplementary Data S4). Meanwhile, Normalized Mutual Information (NMI) was also used to measure the clustering performance (Supplementary Fig. S1, Supplementary Data S4). Moreover, 2 other major criteria (Silhouette score, AMI) were used to evaluate the performance of these 10 repeated experiments of DeepCCI cell cluster model (Fig. 2b and Supplementary Fig. S2). The results of these repeated experiments demonstrated the stability of DeepCCI in cell clustering for scRNA-seq data. Meanwhile, the running time of these approaches across all datasets is being demonstrated (Supplementary Fig. S3).

In addition, we can visualize cell clustering results based on DeepCCI cluster embedding by using the UMAPs (Fig. 2c). By visualizing cell clustering results on UMAPs, one can observe the apparent nearness of cells within the same cluster and separation among different clusters when using embedding provided by the cluster model of DeepCCI (Fig. 2c). Our results indicate that the cluster model of DeepCCI can capture the essential hidden information of cells and make full use of the topological relationships among cells to accurately predict cell clusters.

On top of that, the cluster number is an important parameter for the performance of unsupervised clustering methods. For scRNA-seq data with unknown cell clusters, the cell cluster model of DeepCCI provides a strategy to determine the number of clusters before clustering and the number of clusters is determined by the Louvain algorithm on the cell graph. To evaluate the effectiveness of this strategy, we continued to compare the clustering performance of DeepCCI cluster model with the several tools (scGNN, Seurat, CIDR, RaceID) that with the ability to automatically define the number of clusters on four common datasets (i.e. Chung, Kolodziejczy, Klein, and Zeisel). In comparison with these methods, DeepCCI has achieved better cluster performance on the overwhelming majority of datasets (Supplementary Fig. S4). Especially on the Zeisel dataset, the ARI value of the cluster model of DeepCCI is 0.16 higher than scGNN. Taken together, the GCN we used in DeepCCI cluster model can capture the high-order representations of relationships between cells in scRNA-seq data in the context of graph topology, and apply it to the clustering of scRNA-seq data.

### 3.3 Performance evaluation of interaction model of DeepCCI

**Figure 3.**
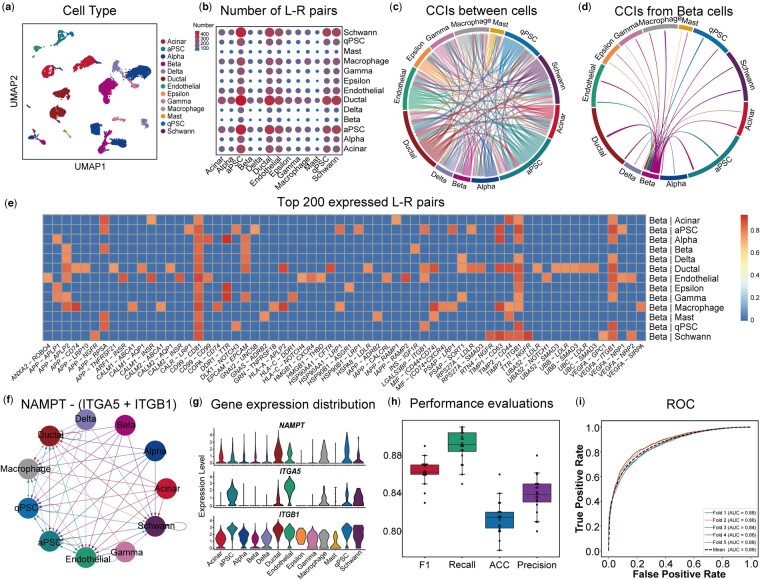
Performance evaluation of cell interaction model of DeepCCI. (a) Benchmarked cell clusters visualized by UMAP for pancreatic islets scRNA-seq dataset. (b) Number of significant L–R pairs between any pair of two cell clusters. (c) Chord plot shows the interactions between all cell clusters. The arrow from source cell cluster point to the target cell cluster and the color of arrow is same as the source cell cluster. (d) Chord plot shows the interactions from beta cell cluster to other cell clusters. (e) Heatmap shows the top 200 expressed L–R pairs between beta cluster and other clusters. The color of the heatmap represents the interaction probability value. (f) Interaction network mediated by the *NAMPT*—(*ITGA5*+*ITGB1*). (g) Gene expression distribution of the *NAMPT* ligand and *ITGA5*, *ITGB1* receptors. (h) Performance evaluations of interaction model of DeepCCI of 5-fold cross valuation for 20 times. (i) The ROC curve of 5-fold cross valuation for DeepCCI interaction model

To present the significant interactions intuitively, we provide several visualization methods (Fig. 3a–g). The bubble plot provides an overview of the number of L–R interactions between cell clusters (Fig. 3b). Also, to show all L–R interactions between cell clusters in another intuitive way, we provide the chord plot (Fig. 3c and d). The arrows from the source cell cluster in chord plot mean that the ligands in the source cluster are connected with the receptors in the target cell clusters. Not only display the interactions for all cell clusters, chord plot also could show the interactions from one specific cell cluster to other clusters. Different colors in the chord plot represent different cell clusters. Figure 3d shows the interactions between Beta cell and other cell clusters. Paracrine communication between Beta cells and non-Beta cells is known to regulate insulin secretion. The Beta cell is electrically excitable and uses changes in membrane potential to couple variations in the blood glucose concentration to stimulate or inhibition of insulin secretion. And then, the heatmaps were used to reveal the detailed L–R pairs with top 200 probability values from the source cluster to target clusters (Fig. 3e).

Moreover, for the L–R pair of interest, the network graph provides an overview of intercellular communication networks, consisting of two components: the cell clusters in the panc8 dataset and the interactions between these clusters (Fig. 3f). The color of the edges is the same as the source cluster. The interaction network for the NAMPT—(ITGA5+ITGB1) pair53 is shown using a network graph. NAMPT—(ITGA5+ITGB1) pair is from Visfatin signaling pathways6 and visfatin was described to be a highly expressed protein with immune cell signaling and nicotinamide adenine dinucleotide (NAD) biosynthetic activity, which is essential for pancreatic beta cell function. Although ITGB1 subunits are expressed in all cell clusters, the cluster that does not express ITGA5 subunits is not the target cluster for the source clusters (Fig. 3g). Taken together, the standards we used to define significant interactions can capture biologically meaningful CCIs from scRNA-seq data.

Further, based on these significant interactions, the interaction model of DeepCCI was constructed (see Section 2). To ensure the generalizability of the interaction model of DeepCCI, we train the model using 5-fold cross-validation. Moreover, 5-fold cross-validation was performed 20 times and 5 criteria (F1, Recall, ACC, Precision, AUC) were used to measure the performance of interaction model of DeepCCI (Fig. 3h and i). Collectively, DeepCCI can identify key features of CCIs within a given scRNA-seq dataset and predict complex intercellular communications in an easily interpretable way.

### 3.4 Independent testing of interaction model of DeepCCI

To investigate whether the interaction model of DeepCCI is applicable to the prediction of CCIs using scRNA-seq data, we used two different datasets (Supplementary Fig. S5a and b). Four criteria are used to evaluate the performance of the model (Supplementary Fig. S5c).

Atopic dermatitis (AD) is a common inflammatory skin disease characterized by immune cell and epidermal abnormalities (He *et al.* 2020). In the human AD dataset, we visualized the predicted L–R pairs between different cell clusters using a bubble plot. The interactions between the cDC1 cell cluster and other clusters in the human AD dataset were displayed in a chord plot (Supplementary Fig. S5d). cDC1 and cDC2 originate from dermal conventional dendritic cells and cDC1s are known for their expression of XCR1 and their ability to present antigens to CD8+ T cells. They play a critical role in immunity against intracellular pathogens and cancer (Xue *et al.* 2020). Fibroblasts (FIBs) are present in various tissues but their characteristics are not well understood. Different subpopulations of fibroblasts with distinct functions have been identified in the skin. FIBs respond to signals from the overlying epidermis, including Wnt-regulated signals, which can influence their behavior and the deposition of the extracellular matrix (ECM) in different parts of the dermis (Driskell and Watt 2015). The intercellular network of the MIF-(CD74+CXCR4) pair is displayed for the human disease skin dataset (Supplementary Fig. S5e–g). MIF is known to be involved in the progression of inflammatory and autoimmune diseases and interacts with various receptors, including CXCR2, CXCR4, and CD74 (Bucala 2013, Morrison and Kleemann 2015). DeepCCI predicted that the CCL19-CCR7 L–R pair is a significant signaling pathway for communication between Inflam.FIB and Inflam.DC, which is consistent with previous experimental findings (He *et al.* 2020, Jin *et al.* 2021).

**Figure 4.**
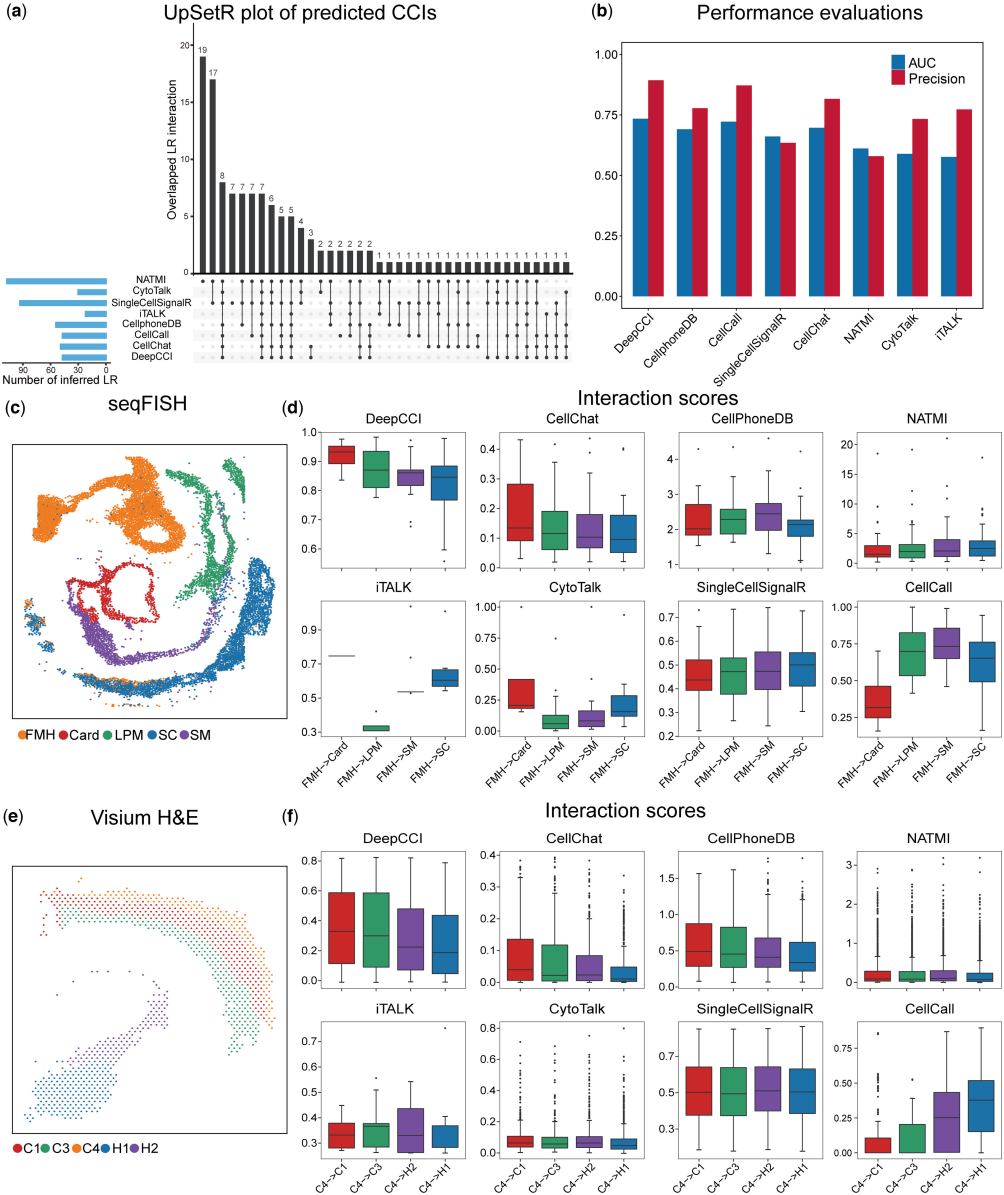
Comparison of the performance of DeepCCI with state-of-the-art methods. (a) UpSetR plot of predicted CCIs from the eight tools of human testicular cells. (b) The AUC and precision of eight CCI predicted methods. (c) Benchmarked cell clusters visualized by UMAP for the seqFISH dataset of mouse organogenesis. (d) Comparison of the interaction strength between spatially adjacent and distant cells for the seqFISH dataset. The interaction strength is calculated by the interaction probability or score. (e) Benchmarked cell clusters visualized by UMAP for 10× Visium spatial transcriptomics dataset of the mouse brain. (f) Comparison of the interaction strength between spatially adjacent and distant cells for 10× Visium spatial transcriptomics dataset of the mouse brain

For the embryonic mouse skin dataset, the interactions of FIB-A cell cluster with other clusters are visualized using a chord plot (Supplementary Fig. S5h). In addition, the network graphs (Supplementary Fig. S5i and j) display the Bmp7-(Bmpr1a+Bmpr2) pair, which is part of the Bone Morphogenetic Protein (BMP) pathway. The BMP pathway, a member of the TGF-beta family of cytokine growth factors, plays a crucial role in development. BMP signaling components have primarily been considered tumor-suppressive, as shown by loss and gain of function studies. Bmp7, a ligand of the TGF-beta family, exhibits tightly regulated expression patterns within the epidermal microenvironment during steady-state and inflammation (Yu *et al.* 2020). When Bmpr2 is dominantly expressed as a negative regulator in a mouse model of breast cancer, it promotes tumor metastasis by creating a paracrine inflammatory microenvironment (Owens *et al.* 2012). The heatmap (Supplementary Fig. S5k) displays the top 200 probability values of predicted L–R pairs between the DC cluster and other cell clusters in the embryonic mouse skin dataset. Dendritic cells (DCs) play a critical role in activating both the innate and adaptive immune systems during acute tissue injury (Nace *et al.* 2011). They serve as messengers between the innate and adaptive immune responses, contributing to every phase of wound healing. Our predicted L–R interactions indicate strong intercellular communication between the DC cluster and other cell clusters, including the Notch signaling ligands Dlk1, as well as members of the collagen family such as Col1a1, Col1a2, and Col4a1 (Macedo and Kaiser 2019) (Fig. 4k). The overexpression of collagen ligands on DCs and their interaction with receptors on other cell types highlight the significant role of DCs in sensing tissue damage and promoting skin wound repair. In summary, DeepCCI demonstrates its ability to accurately identify meaningful interactions from complex and sparse scRNA-seq data, even across datasets generated by different platforms.

### 3.5 Comparison of DeepCCI with state-of-the-art methods for predicting CCIs

We systematically compared DeepCCI with seven state-of-the-art methods (SingleCellSignalR, iTALK, CellPhoneDB, CellChat, CellCall, CytoTalk, and NATMI) that offered thresholds for communication scores and all the methods were applied to infer CCIs using their own default parameters.

First, we compared the performance of DeepCCI with seven CCI prediction methods on the dataset of human testicular cells (see Section 2). DeepCCI identified 47 CCIs from STs to SSCs and SingleCellSignalR, iTALK, CellPhoneDB, CellChat, CellCall, CytoTalk, and NATMI identified 93, 22, 54, 49, 47, 30, and 107 CCIs by their own default cut-offs (Fig. 4a, see Supplementary Data S6 for details). Over 89% of DeepCCI-identified CCIs (42/47) have been reported to be involved in spermatogenesis and the high literature support rate is superior to other tools. DeepCCI achieved the highest AUC and precision on the scRNA-seq dataset of human testicular (Fig. 4b). These results suggest that DeepCCI has the ability to discover biologically meaningful CCIs from scRNA-seq data.

Next, we made use of the single-cell resolutions of seqFISH dataset to identify both the spatially adjacent cell types and spatially distant cell types to obtain the interaction predictions. According to the spatial location of these cells, the Crad cells are spatially adjacent to FMH and the SC cells are spatially distant to FMH. Also, LPM cells are slightly closer to FMH in spatial position than SC cell type (Fig. 4c and Supplementary Fig. S6). We compared the interaction strength (probabilities or scores) between each cell cluster predicted by DeepCCI with the other seven methods (Fig. 4d). We found that DeepCCI consistently captures stronger interactions in spatially adjacent cells than in distant cells of the interaction probabilities. Meanwhile, DeepCCI also performed well at discriminating spatially adjacent from distant cells and other tools failed to capture stronger interactions in spatially adjacent cells as compared to spatially distant cells.

And then, we conducted a similar analysis with 10× Visium spatial transcriptomics dataset of the mouse brain. From the spatial distribution of these cell types, we can easily find the spatial relationship of C4 cell type with other cell types. The C1 and C3 cell types are spatially adjacent to C4 cell type and the H1 and H2 cell types are spatially distant to C4 cell type. In addition, compared with H1 cell type, H2 is slightly closer to C4 cell type in spatial position (Fig. 4e and Supplementary Fig. S7). We found a clear association between the predicted CCIs and the spatial adjacency of their corresponding cell types for DeepCCI, CellChat, and CellPhoneDB, while the other methods showed inconsistent trends (Fig. 5f). Moreover, compared with CellChat and CellPhoneDB, the predicted interactions of DeepCCI from C4 cell type to C3 cell and H2 cell are better to reflect the spatial relationship between C4 and these two cell types. Together, our analyses show that DeepCCI performs well at predicting biologically meaningful interactions in spatially adjacent cells than in distant cells from spatial transcriptomics datasets. In addition, the chart illustrating the running time of these computationally CCI predicted methods is included (Supplementary Fig. S8).

### 3.6 De novo prediction of CCIs using DeepCCI

Firstly, we clustered the cells to nine groups using the cluster model of DeepCCI and ARI was used to measure the clustering performance. The ARI value reached 0.71 and the visualization result is shown using UMAP (Supplementary Fig. S9a and b). From the visualization result, we can see that the annotated labels are roughly the same as the benchmarked labels. Finally, we identified the significant interactions jointly using these predicted cell clusters and the interaction model of DeepCCI.

For ease of comparison, we set the interaction results defined by benchmarked labels and three statistical methods (see Section 2) as the high-confidence interactions. The chord plots illustrate the interactions between the B cell cluster and other cell clusters under the two conditions (Fig. 6c). In addition, the network graph illustrates the interplay facilitated by MIF—(CD74+CD44), while the gene expression of L–R pair for all clusters is elaborated upon (Supplementary Fig. S9e and f). MIF activates the ERK 1/2 MAPK signaling pathway by binding to the CD74/CD44 complex, which seems to play a supportive role in immune cell stimulation (Hoshina *et al.* 2008). The phosphorylation of MAPK, upon CD74 engagement, necessitates the co-expression of CD44, a catalyst for the Src tyrosine kinase (Hoshina *et al.* 2008). To visually represent the interactions mediated by MIF—(CD74+CD44), the probability values of interactions between cell clusters are presented (Supplementary Fig. S9g and h). When comparing these results, one can observe that the predicted interaction network mediated by MIF—(CD74+CD44) generally aligns with the interactions of high-confidence. Our findings affirm that DeepCCI accurately predicts crucial CCIs within similar cell clusters. Moreover, heatmaps portray the probability of interaction for L–R pairs originating from the B cell cluster to other clusters, both for high-confidence interactions and predicted interactions (Supplementary Fig. S9i and j). The resulting heatmap of predicted interactions closely resembles that of the high-confidence interactions, demonstrating that DeepCCI effectively highlights significant interactions. CD2 and CD48 enhance initial T cell signaling, and CD2 is necessary for the association of CD48 with the TCR and CD3. Furthermore, a prior study has demonstrated that the interactions between CD48 and CD2 contribute to both the priming and effector functions of CD8+ T cells. Hence, the predicted CD48–CD2 interaction by DeepCCI, from B cells to Naïve and CD8+ T cells, holds biological significance. When compared to high-confidence interactions defined by multiple statistical methods, the DeepCCI interaction model successfully predicts significant interactions in both general and specific scenarios. In essence, DeepCCI possesses the capability to comprehensively identify CCIs from single-cell RNA sequencing (scRNA-seq) data. Even in scRNA-seq data lacking cell labels, DeepCCI can identify biologically meaningful cell clusters and unveil significant interactions between these clusters.

## 4 Conclusion

It is still a fundamental challenge to identify intercellular communications from high-volume, high-sparsity, and noisy scRNA-seq data. We described DeepCCI, a tool to help users identify cell clusters and CCIs from scRNA-seq data. To cluster cells from scRNA-Seq data, DeepCCI maps all cells in a joint low-dimensional embedding space using GCN-based deep neural networks. The learned joint embedding can be treated as the high-order representations of relationships between cells in scRNA-Seq data. In comparison to several state-of-the-art methods for scRNA-seq clustering, DeepCCI achieved the best clustering accuracy across various datasets. To explore more biologically meaningful intercellular interactions from scRNA-seq data, we constructed the most comprehensive set of L–R pairs. DeepCCI is the first approach to explore intercellular interactions using the deep learning framework from scRNA-seq data. The key innovations of DeepCCI are incorporating global propagated topological features between L–R pairs through GCNs, together with integrating gene expression of cell pairs in the prediction process for intercellular interactions. DeepCCI allows users to quickly identify significant interactions based on single-cell expression data and visualize overall CCI networks under different conditions.

Although we defined the significant CCIs based on the most comprehensive L–R pairs database and several publicly available statistical methods, systematic evaluation of predicted CCIs is challenging due to the lack of ground truth. Recent advances in spatially resolved transcriptomic techniques offer an opportunity to explore the spatial organization of cells in tissues (Atta and Fan 2021). Integrating scRNA-seq and spatial transcriptomics data may therefore increase our understanding of the roles of specific cell subpopulations and their interactions in development, homeostasis and disease (Longo *et al.* 2021). In the future, we will continue to enhance DeepCCI to support the integration of single-cell multi-omics sequencing (scMulti-seq) data and incorporate spatial transcriptomics to make the analyses more explainable. We plan to develop a more user-friendly software system based on our DeepCCI model, together with modularized analytical functions in support of data format standardization, quality control, data integration, multi-functional scMulti-seq analysis, performance evaluation, and interactive visualization.

## Supplementary Material

btad596_Supplementary_Data
