# Supplementary material for: DeepCCI: a deep learning framework for identifying cell–cell interactions from single-cell RNA sequencing data
Source: Bioinformatics. 2023 Sep 23;39(10):btad596. doi: 10.1093/bioinformatics/btad596 (PMC10558043; doi:10.1093/bioinformatics/btad596)
Supplement: btad596_Supplementary_Data [file btad596_supplementary_data.zip › Supplementary figures(1).docx]

**Supporting Information for**

DeepCCI: a deep learning framework for identifying cell-cell interactions from single-cell RNA sequencing data

Wenyi Yang^1,#^, Pingping Wang^1,#^, Meng Luo^1^, Yideng Cai^1^, Chang Xu^1^, Guangfu Xue^1^, Xiyun Jin^1^, Rui Cheng^1^, Jinhao Que^1^, Fenglan Pang^1^, Yuexin Yang^1^, Huan Nie^1^, Qinghua Jiang^1,*^, Zhigang Liu^2,*^, Zhaochun Xu^1,*^

^1^School of Life Science and Technology, Harbin Institute of Technology, Harbin, China

^2^ Affiliated Foshan Maternity & Child Healthcare Hospital, Southern Medical University, Guangzhou, China

*To whom correspondence should be addressed.

**This PDF file includes:**

Figs. S1 to S10

**Supplementary Figures**

**
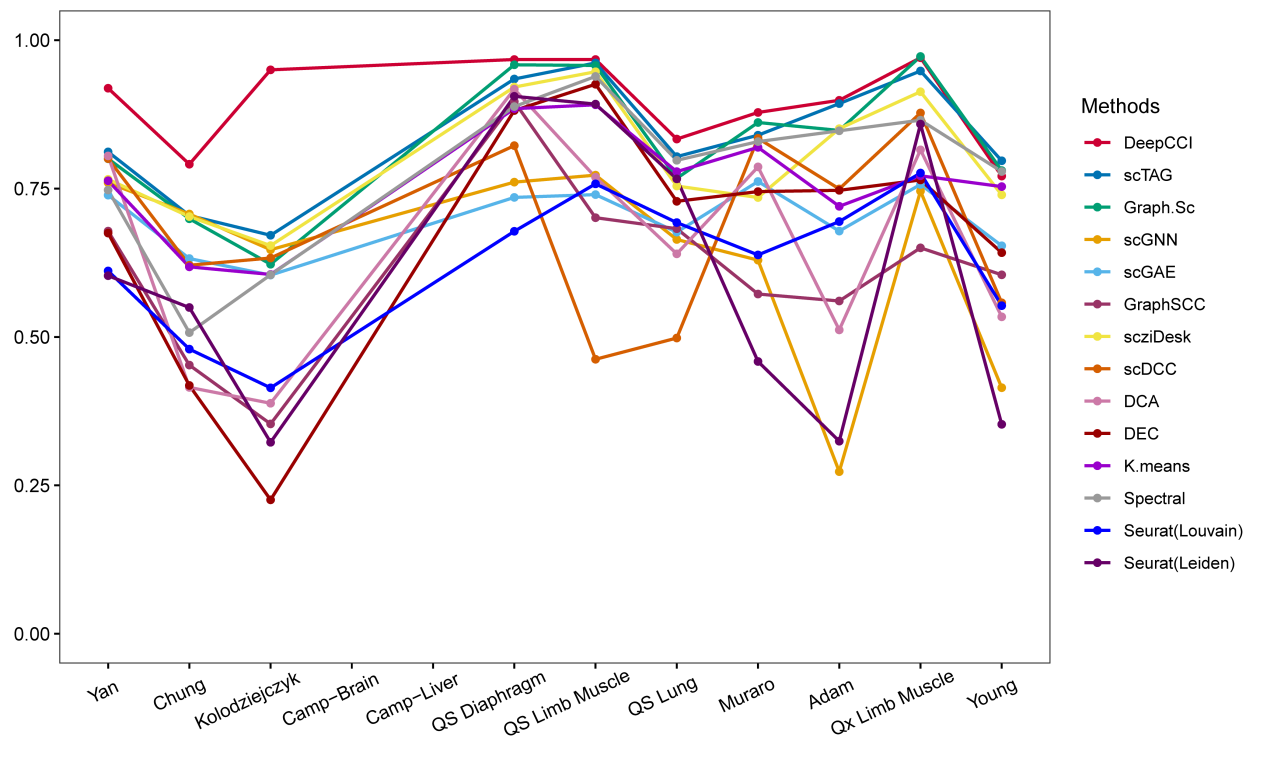
**

**Fig. S1. Performance evaluation of cell cluster model of DeepCCI.**

Comparison of NMI among cell cluster model of DeepCCI and 13 state-of-the-art methods using 12 real-world scRNA-seq datasets.

**
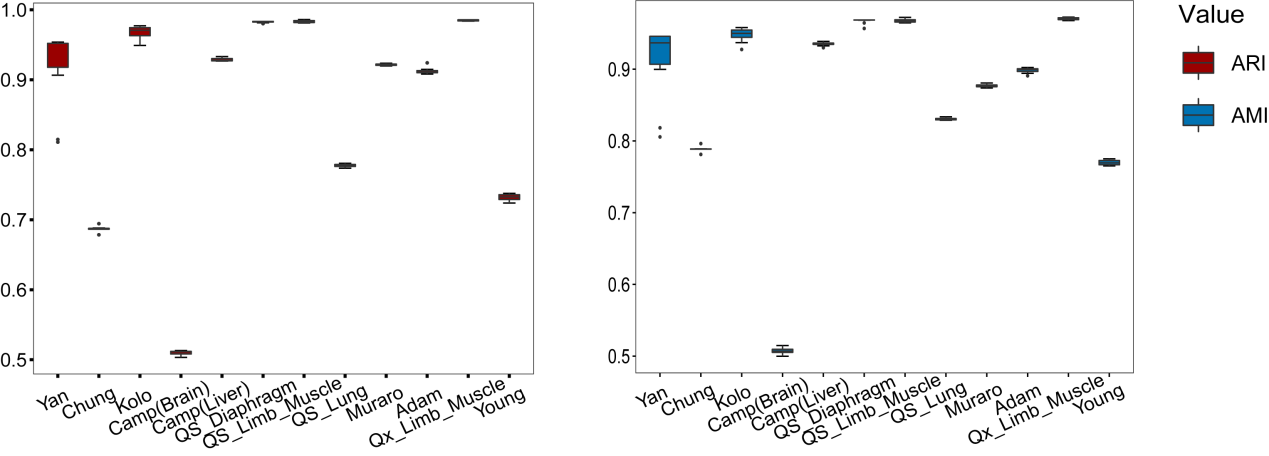
**

**Fig. S2. Performance evaluations for the stability of the cell cluster model of DeepCCI.**

Performance evaluations of the cell clustering by repeating cell cluster model of DeepCCI 10 times on 12 scRNA-seq datasets.

**
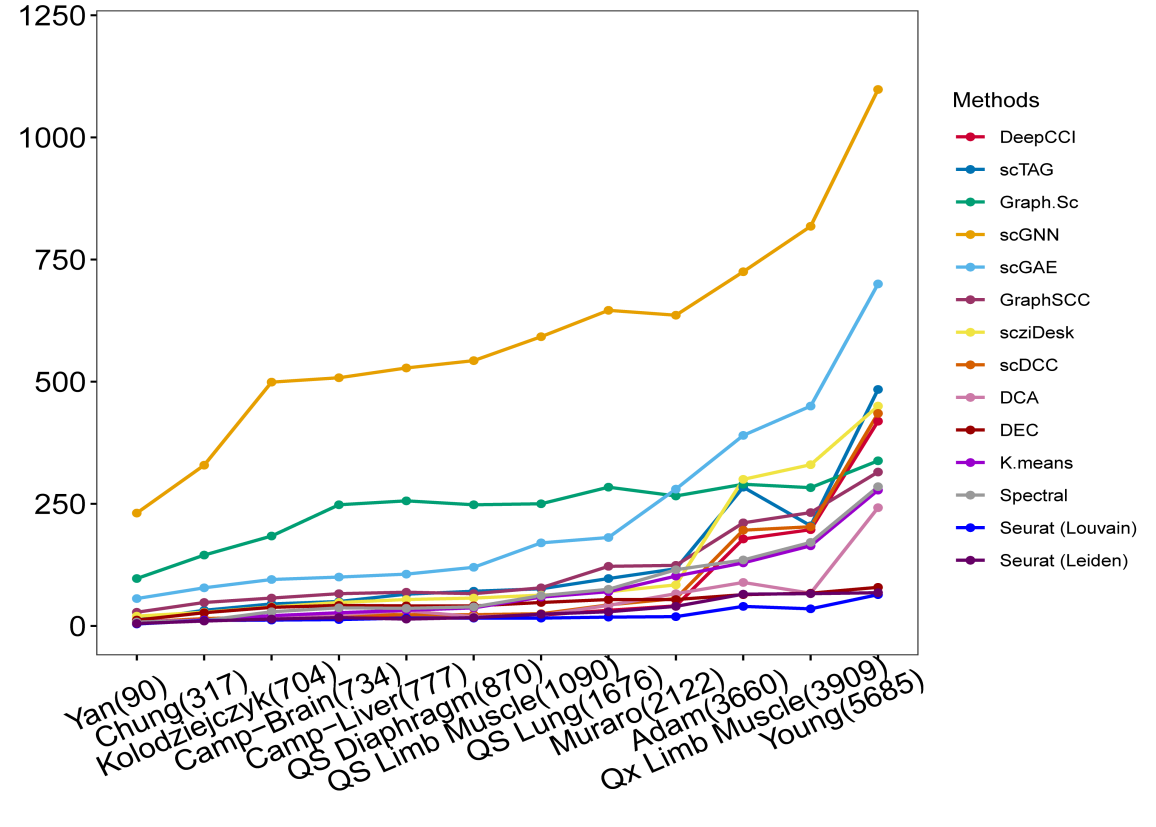
**

**Fig. S3. Running time of 14 clustering methods on 12 scRNA-seq datasets.**

Running time of all methods on 12 scRNA-seq datasets.

**
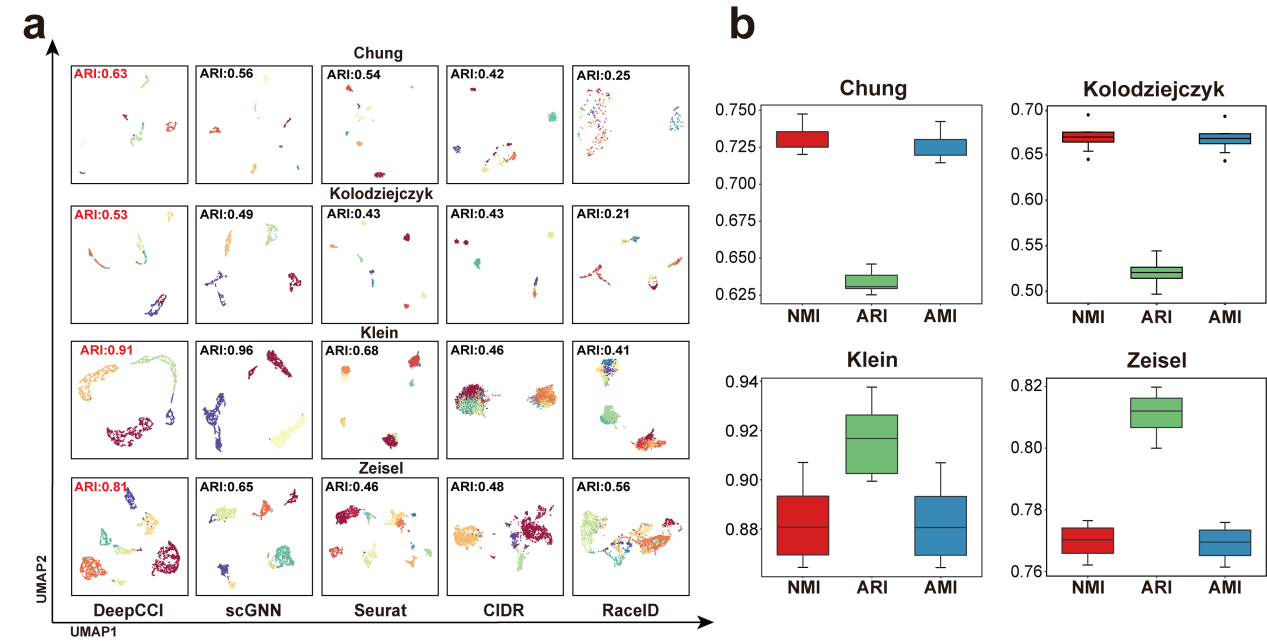
**

**Fig. S4. Comparison with several methods for cell clustering.**

**a**, Comparison of UMAP visualizations on Chung, Kolodziejczy, Klein and Zeisel datasets. **b**, Performance evaluations of the cell clustering by repeating cell cluster model DeepCCI 10 times on the same four scRNA-seq datasets.

**
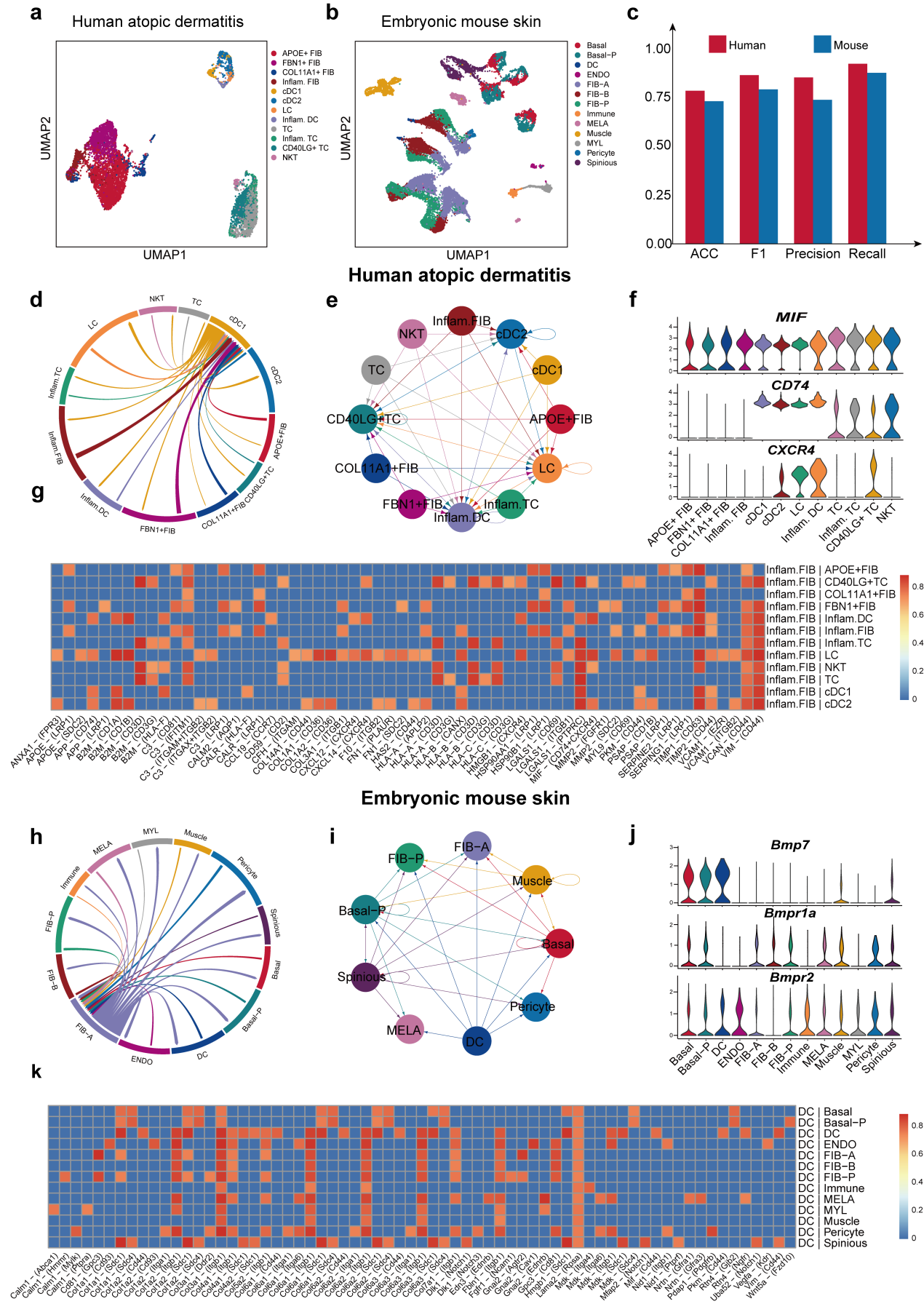
**

**Fig. S5.** Independent testing of interaction model of DeepCCI. **a-b**, Benchmarked cell clusters visualized by UMAP. **a** represents the human atopic dermatitis dataset and **b** represents embryonic mouse skin dataset. **c**, Performance of interaction model of DeepCCI on human and mouse scRNA-seq datasets. **d,** Chord plot shows the predicted interactions from cDC1 to other cell clusters for the human atopic dermatitis dataset. **e**, The predicted interaction network mediated by *MIF* - (*CD74* + *CXCR4*). **f**, Gene expression distribution of the *MIF*, *CD74* and *CXCR4*. **g**, Heatmap shows the top 200 expressed predicted L-R pairs for Inflam.FIB to other clusters. **h,** Chord plot shows the predicted interactions from FIB-A cell cluster to other clusters for the embryonic mouse skin dataset. **i**, Interaction network mediated by *Bmp7* - (*Bmpr1a* + *Bmpr2*) pair. **j**, Gene expression distribution of the *Bmp7*, *Bmpr1a* and *Bmpr2*. **k**, Heatmap shows the top 200 expressed predicted L-R pairs for DC to other clusters.


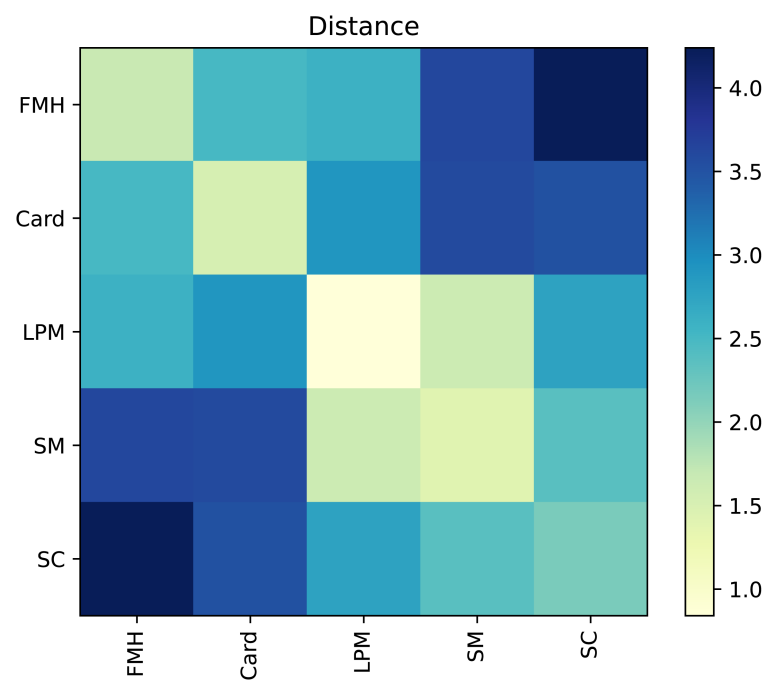


**Fig. S6. The distance between five cell types for seqFISH dataset of mouse organogenesis.**

The plot of distance between 5 cell types for seqFISH dataset of mouse organogenesis.


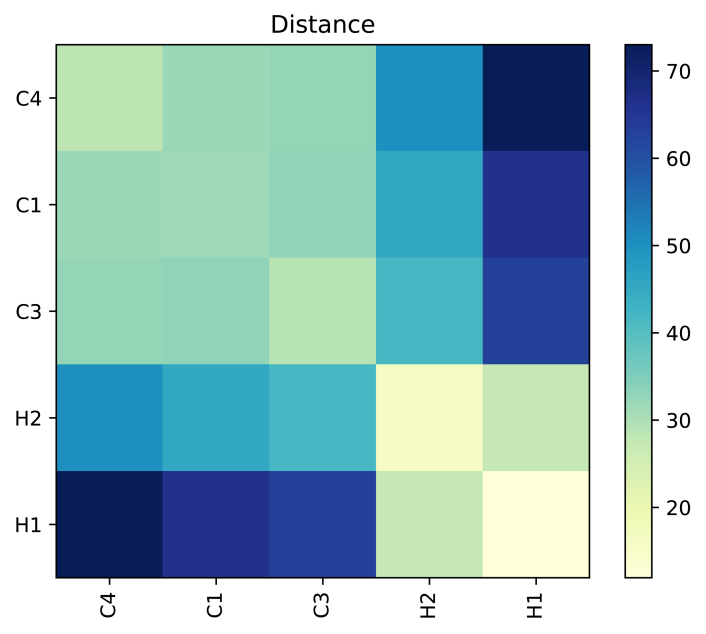


**Fig. S7. The distance between five cell types for 10X Visium spatial transcriptomics dataset of the mouse brain.**

The plot of distance between 5 cell types for 10X Visium spatial transcriptomics dataset of the mouse brain.


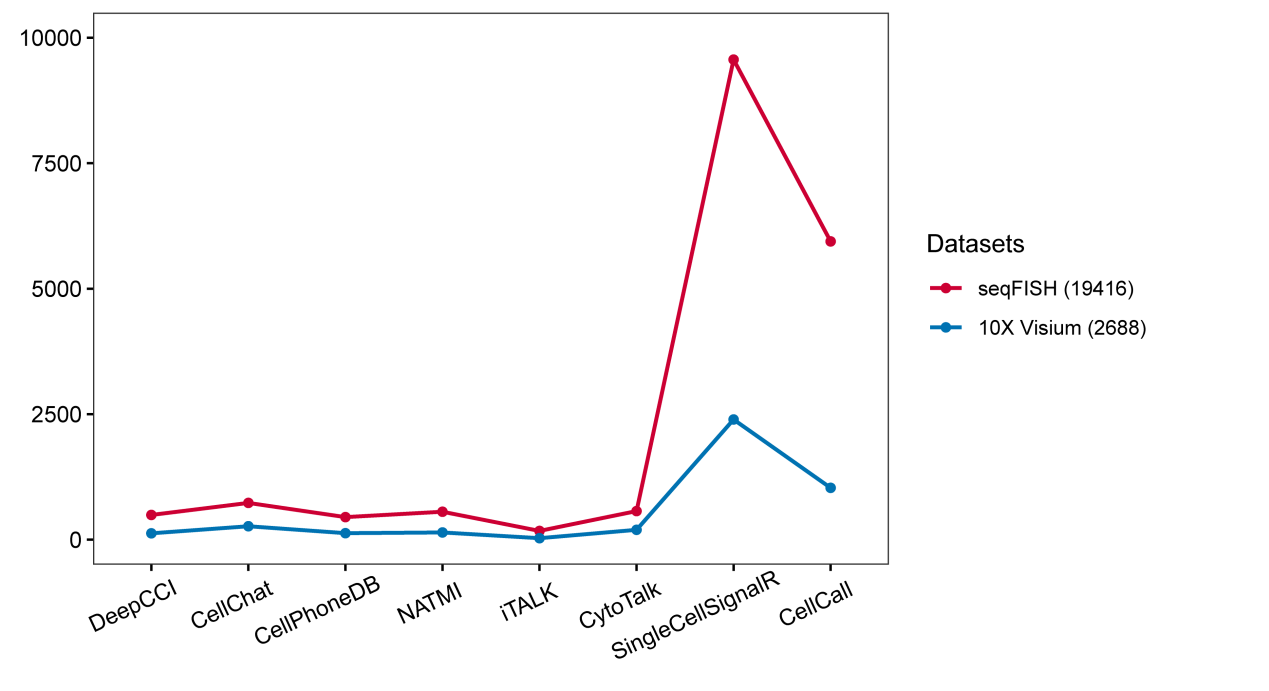


**Fig. S8. Running time of 8 CCI predicted methods on2 spatial transcriptomics datasets.**

The plot of running time for CCI prediceted methods on seqFISH dataset of mouse organogenesis and 10X Visium spatial transcriptomics dataset of the mouse brain.


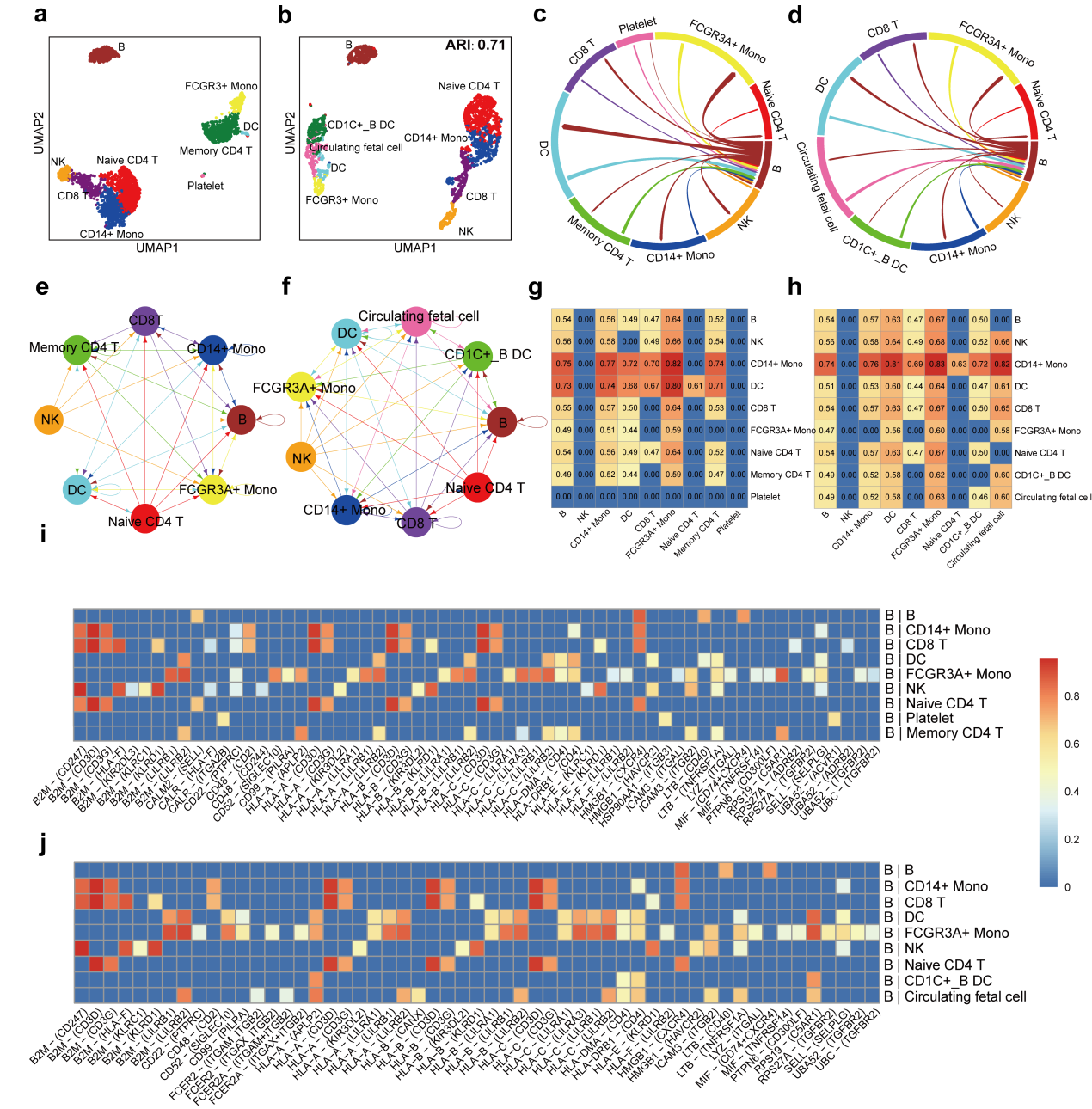


**Fig. S9. **Comparison analysis between high-confidence and predicted interactions**.** **a,** Benchmarked cell labels of PBMC3K dataset visualized by UMAP. **b,** Predicted cell labels of PBMC3K dataset visualized by UMAP. **c**, High-confidence interactions between B cell cluster and other cell clusters. **d,** Predicted interactions from B cell cluster to other clusters. **e,** High-confidence interaction network mediated by *MIF* - (*CD74* + *CD44*) pair. **f**, High-confidence interaction network mediated by *MIF* - (*CD74* + *CD44*) pair. **g,** Interaction probability values of high-confidence interaction between all clusters under the *MIF* - (*CD74* + *CD44*) pair. **h,** Interaction probability values of predicted interactions between all clusters under the *MIF* - (*CD74* + *CD44*) pair. **i,** Heatmap of high-confidence L-R pairs with the interaction probability value for B cell cluster to other clusters. **j,** Heatmap of predicted L-R pairs with the interaction probability value for B cell cluster to other clusters.


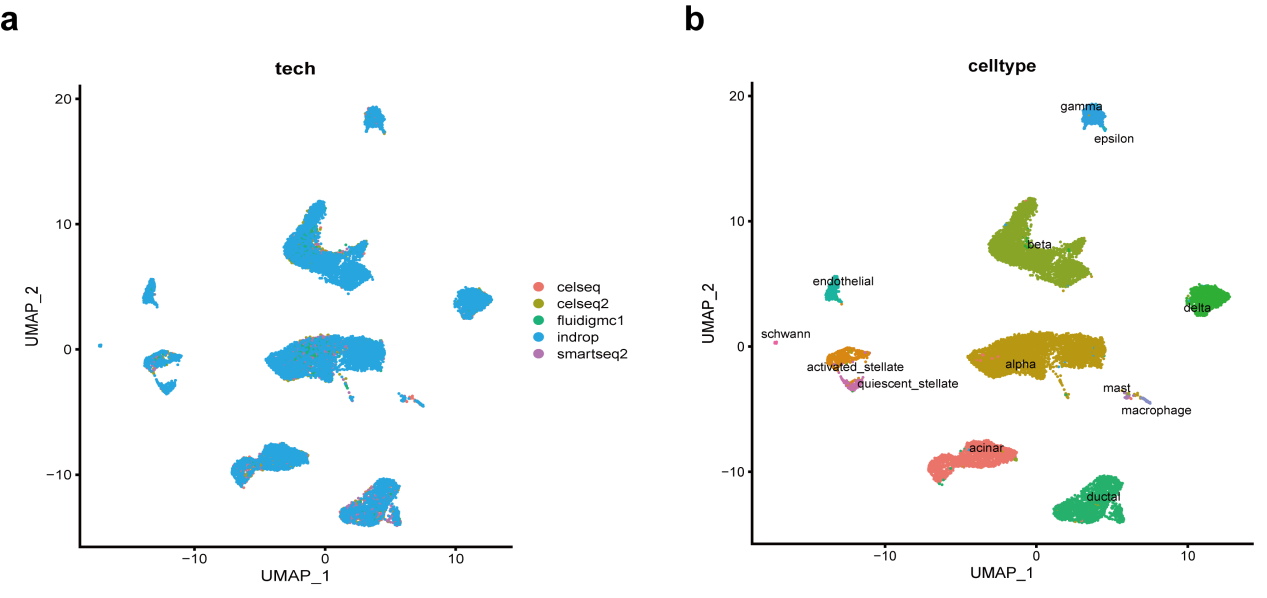


**Fig S10. The batch effect correction of scRNA-Seq data of pancreatic islets.**
